# Editorial: Association of novel anthropometric indexes with metabolic syndrome and beyond, volume II

**DOI:** 10.3389/fendo.2023.1138022

**Published:** 2023-01-18

**Authors:** Nami Mohammadian Khonsari, Patricia Khashayar, Roya Kelishadi, Ozra Tabatabaei-Malazy, Mostafa Qorbani

**Affiliations:** ^1^ Non-Communicable Diseases Research Center, Alborz University of Medical Sciences, Karaj, Iran; ^2^ Center for Microsystems Technology, Imec & Ghent University, Gent, Belgium; ^3^ Department of Pediatrics, Child Growth and Development Research Center, Research Institute for Primordial Prevention of Non-Communicable Disease, Isfahan University of Medical Sciences, Isfahan, Iran; ^4^ Non-Communicable Diseases Research Center, Endocrinology and Metabolism Population Sciences Institute, Tehran University of Medical Sciences, Tehran, Iran

**Keywords:** obesity, novel anthropometric indexes, metabolic syndrome, body mass index, visceral fat, cardiometabolic risk factors, neck circumference, waist circumference

Obesity has become a primary global health concern. One cannot deny the role of the increasing prevalence of obesity in the surging trend of cardiometabolic conditions such as type 2 diabetes (T2DM), hypertension (HTN), dyslipidemia, insulin resistance (IR), and many other conditions (1, 2). Hence due to the importance of the subject, we have tried to provide a more accurate definition for obesity to prevent, find and treat the affected cases more effectively. Body Mass Index (BMI), which is the weight/square of height, has been used to define obesity since the 70s; however, despite many benefits, it suffers from certain flaws, as it measures excess weight only rather than excess fat. Thus new anthropometric measures have recently emerged to define obesity whirls overcoming the known flaws of BMI (3, 4). In volume II of the Research Topic entitled “*Association of Novel Anthropometric Indexes with Metabolic Syndrome and Beyond*” similar to the previous volume, the links between these novel anthropometric indices, obesity, and cardiometabolic risk factors have been evaluated. Overall, most of the researches included in the current Research Topic were from China and the US. These studies assessed the role of new anthropometric measurements for early detection of obesity, metabolic syndrome (MetS), IR and their association with less studied comorbidities such as renal function.

Despite the virtues of the BMI measurement, it is incapable of distinguishing lean and fat body mass from one another; another critical flaw of BMI measurement is its various classifications based on age, ethnicity, and sex. An article by Al-Hazzaa et al. addressed this issue by comparing three BMI classifications on 2169 Saudi children. They reported the prevalence of overweight and obesity to be 31.1% based on the Saudi national growth references, 31.7% for the International Obesity Task Force, and 38.0% for the World Health Organization. Regardless of the high prevalence of overweight and obesity in these children, the inconsistency of BMI measurements in estimating the prevalence of obesity based on various classifications is undeniable. Hence the use of novel anthropometric measurements to overcome these flaws is gaining more interest. In this regard, body composition parameters are shown to better reflect the association between obesity and metabolic disorders (5). In a study by Qi et al. on 12148 US adults, body composition, also known as adiposity, was assessed using dual-energy X-ray Absorptiometry (DXA). They found a positive association between the upper limb, torso, and whole-body fat mass percentage and odds of developing HTN, hypercholesterolemia, and T2DM. They also noted that increased adiposity was associated with higher risk of metabolic conditions in men than in women.

To define the association between obesity and vascular disorders such as ischemic stroke and atherosclerosis, the measurement of waist circumference (WC) is more recommended than BMI since it represents visceral fat accumulation (6); similarly, neck circumference (NC), despite being simple to measure, is significantly associated with adiposity. A study by Ren et al. on 431 stroke patients reported increased WC alongside with hypertriglyceridemia. Otherwise known as hypertriglyceridemia waist phenotype (HTWP), the condition was associated with higher odds of moderate to severe small artery occlusion strokes. Similarly, Fodra Fojas et al. used NC as a surrogate for body composition and assessed its association with dysglycemia, MetS, and non-alcoholic fatty liver disease (NAFLD) in an Emirati population. They found NC to be associated with dysglycemia, MetS, and NAFLD. Every one cm increase in NC was also shown to significantly increase the hazard of cardiovascular risk score by 15%. This highlights the importance of developing new measures with robust predictive properties. Taking these into account, Liu et al. in a study conducted on 721 overweight and obese Chinese participants developed new equations to estimate visceral obesity. The assessment of visceral obesity is of great importance since it is associated with genes linked with inflammation, oxidative stress, and cytokine dysregulation among others. They calculated the visceral fat area (VFA) to be equal to 3.7×age+2.4×WC+5.5×NC-443.6 in men and 2.8×age+1.7×WC+6.5×NC-367.3 in women with good predictive properties for visceral obesity.

Five studies from this topic focused on the laboratory indices and their associations with MetS. The use of laboratory indices can accompany anthropometric measurements for a better assessment, especially in those without visible adiposity. Both viral hepatitis and fatty liver disease can result in abnormal levels of alanine aminotransferase (ALT) and aspartate aminotransferase (AST) (7). A study by Lin et al. on 2416 Taiwanese participants showed the ALT to AST ratio of higher than 1 to be a simple yet reliable index for MetS regardless of the presence of underlying viral hepatitis.

Due to the concordance of chronic inflammation and MetS, Wang et al. studied novel pro-inflammatory indices and their association with MetS in newly diagnosed T2DM patients. They assessed the ratio of monocyte (one of the key cells in the innate immune system) to high-density lipoprotein cholesterol (MHR) and monocyte to apolipoprotein A1 (MAR), and found them to be correlated with the metabolic risk factors such as triglycerides, high-density lipoprotein cholesterol (HDL-C), systolic and diastolic blood pressure, uric acid, IR, BMI, and WC. Moreover, MHR and MAR values above 3.57 × 10^8^/mmol and 3.95 × 10^8^/g, respectively, were shown to have a higher than 70% sensitivity and specificity in identifying MetS. A study by Duan et al. on 1452 Chinese participants assessed the predictive capacity of BMI, lipid accumulation product (LAP), body roundness index (BRI), Chinese visceral adiposity index (CVAI), body adiposity index (BAI), abdominal volume index (AVI), triglyceride glucose index (TYG), and visceral adiposity index (VAI). Interestingly, they found that the lipid-based set of LAP, TYG, CVAI and VAI had a higher predictive value than the anthropometry-based set of BMI, BRI, AVI and BAI, indicating their potential capacity as screening tools for MetS. Another laboratory index to predict MetS is hyperuricemia. While elevated serum levels of urate are associated with a broad spectrum of conditions, excess fat increases the production of hyperuricemia by affecting the liver. Thus, the assessment of hyperuricemia can illustrate a better view of the body’s metabolic status as demonstrated by Wang et al. They showed that hyperuricemia is positively associated with increased TYG, TYG to HDL-C ratio, and IR. Another study by Zhao et al. on 14078 hypertensive patients also found a significant association between a novel anthropometric measure for obesity, called “weight-adjusted-waist index” (WWI), and hyperuricemia. This novel measure can distinguish between fat and muscle mass, reflecting central obesity. They found that every one-unit increase in the WWI increases the odds of developing hyperuricemia by 37% and 35% in men and women, respectively ((OR: 1.37; 95%CI: 1.25, 1.49) (OR: 1.35; 95%CI: 1.26, 1.45)). Thus, certain anthropometric measures and the laboratory indices together are believed to be intuited, whereas novel anthropometric measurements can illustrate some degree of the individual’s metabolic status.

There is a notable association between obesity, especially central obesity, and chronic kidney dysfunction (8). In this regard, an article by Zhang et al. evaluated the association between “A body shape index” (ABSI), which is a marker of abdominal obesity and IR, and elevated “urinary albumin to creatinine ratio” (UACR) that is a marker of early kidney injury. This study, which consisted of 40726 adults with no primary kidney diseases, assessed the aforementioned ratio and its correlation with the adverse effects of visceral obesity on kidney function. Higher ABSI values are associated with UACR values higher than or equal to 30 mg/g. This finding is of particular importance since it indicates urinary workups can also be used for obesity risk assessment. It also highlighted the effects of obesity on renal function. Another study by Li et al. on 10858 US participants evaluated the association between body fat distribution and renal stones. This study used the Android to Gynoid ratio (A/G) obtained by DXA to represent visceral fat. They found that higher A/G ratio significantly increased the risk of renal stones among all US ethnic groups and sexes. Another study by Shen et al. studied this matter further by evaluating the association between the Metabolic Score for Insulin Resistance (METS-IR) and renal stones. In this study, conducted on 30612 adults, a significantly positive association was reported between METS-IR and renal stones. Wang et al. also found METS-IR to be associated with gallbladder stones, with every unit increase in METS-IR increasing the odds ratio of gallbladder stone by 3.3% (OR: 1.033, 95% CI (1.0258, 1.0403)).

Last but not least, the final article in this issue was a systematic review evaluating the association between the allostatic load (AL) mediators and MetS. The adaptive response mechanism to chronic stress with the aim of restoring the physiological stability is known as allostasis. This mechanism is mediated by the autonomic nervous system (ANS), the hypothalamic–pituitary–adrenal axis (HPA), the hypothalamic–pituitary–thyroid axis (HPT), somatotropic axes, the gonadal axis (HPG), the metabolic and immune system. AL index consists of various biomarkers that reflect the activity of the aforementioned axes. Two of the assessed biomarkers are dehydroepiandrosterone sulfate (DHEAS; a functional HPA axis antagonist) and cortisol. The systematic review concluded that MetS is associated with higher serum, salivary, hair, and urinary cortisol levels and lower levels of DHEAS.

To conclude, the articles included in this Research Topic points out the importance of new anthropometric measurements since obesity and MetS not only affect the cardiovascular system but also adversely affects the renal function and various functions involved in homeostasis. Proper anthropometric measurements can also give us a notion of the individual’s current metabolic status and improve the risk assessment of various comorbidities. Laboratory workups alongside anthropometric measurements, therefore, can greatly help with the risk assessments.

## Author contributions

All authors listed have made a substantial, direct, and intellectual contribution to the work and approved it for publication.

## References

[1] NCD Risk Factor Collaboration (NCD-RisC). Worldwide trends in body-mass index, underweight, overweight, and obesity from 1975 to 2016: A pooled analysis of 2416 population-based measurement studies in 128·9 million children, adolescents, and adults. Lancet (2017) 390(10113):2627–42. doi: 10.1016/S0140-6736(17)32129-3 PMC5735219 29029897

[2] Aslibekyan S Garvey WT. Obesity and cardiometabolic disease — more than meets the eye. Nat Rev Endocrinol (2017) 13(10):566–8. doi: 10.1038/nrendo.2017.112 28862269

[3] Khonsari NM Khashayar P Shahrestanaki E Kelishadi R Nami SM Heidari-Beni M. Normal weight obesity and cardiometabolic risk factors: A systematic review and meta-analysis. Front Endocrinol (Lausanne) (2022) 13:857930. doi: 10.3389/fendo.2022.857930 35399938 PMC8987277

[4] Payab M Qorbani M Shahbal N Motlagh ME Hasani-Ranjbar S Zahedi H. Association of anthropometric indices with metabolic phenotypes of obesity in children and adolescents: The CASPIAN-V study. Front Endocrinol (Lausanne) (2019) 10:786. doi: 10.3389/fendo.2019.00786 31849834 PMC6902658

[5] Bosello O Vanzo A. Obesity paradox and aging. Eat Weight Disord (2021) 26(1):27–35. doi: 10.1007/s40519-019-00815-4 31865598

[6] Winter Y Rohrmann S Linseisen J Lanczik O Ringleb PA Hebebrand J. Contribution of obesity and abdominal fat mass to risk of stroke and transient ischemic attacks. Stroke (2008) 39(12):3145–51. doi: 10.1161/STROKEAHA.108.523001 18703800

[7] Yan LB Liao J Han N Zhou LY Wang XE Wang YJ. Association between hepatitis b virus infection and metabolic syndrome in southwest China: A cross-sectional study. Sci Rep (2020) 10(1):6738. doi: 10.1038/s41598-020-62609-4 32317690 PMC7174346

[8] Obermayr RP Temml C Knechtelsdorfer M Gutjahr G Kletzmayr J Heiss S. Predictors of new-onset decline in kidney function in a general middle-european population. Nephrol Dial Transplant (2008) 23(4):1265–73. doi: 10.1093/ndt/gfm790 18039642

